# Double-Antibody Sandwich Immunoassay and Plasmonic Coupling Synergistically Improved Long-Range SPR Biosensor with Low Detection Limit

**DOI:** 10.3390/nano11082137

**Published:** 2021-08-22

**Authors:** Jianying Jing, Kun Liu, Junfeng Jiang, Tianhua Xu, Shuang Wang, Jinying Ma, Zhao Zhang, Wenlin Zhang, Tiegen Liu

**Affiliations:** 1 School of Precision Instruments and Opto–Electronics Engineering, Tianjin University, Tianjin 300072, China; jingjianying@tju.edu.cn (J.J.); jiangjfjxu@tju.edu.cn (J.J.); xutianhua@tju.edu.cn (T.X.); sarahwang@tju.edu.cn (S.W.); majinying@tju.edu.cn (J.M.); zhangzhao0905@tju.edu.cn (Z.Z.); 2019202006@tju.edu.cn (W.Z.); tgliu@tju.edu.cn (T.L.); 2 Key Laboratory of Opto–Electronics Information Technology, Ministry of Education, Tianjin University, Tianjin 300072, China; 3 Tianjin Optical Fiber Sensing Engineering Center, Institute of Optical Fiber Sensing, Tianjin University, Tianjin 300072, China

**Keywords:** nanophotonics, plasmonic detection, nanocomposite material, double-antibody sandwich immunoassay, plasmonic coupling between nano gold, low limit of detection

## Abstract

A long-range surface plasmonic resonance (LR-SPR) biosensor modified with double-antibody sandwich immunoassay and plasmonic coupling is demonstrated for human-immunoglobulin G detection with a low limit of detection (LOD). The double-antibody sandwich immunoassay dramatically changes the average refractive index of the medium layer on the sensor surface. The near-field electron coupling between the localized surface plasmon and the long-range surface plasmon leads to a significant perturbation of the evanescent field. The large penetration depth and the long propagation distance of the long-range surface plasmonic waves facilitate the LR-SPR sensor in the detection of biological macromolecules. The unique light absorption characteristic of the nanocomposite material in the sensor provides the in situ self-compensation for the disturbance. Therefore, besides the inherent advantages of optical fiber sensors, the developed biosensor can realize the detection of biomolecules with high sensitivity, low LOD and high accuracy and reliability. Experimental results demonstrate that the LOD of the biosensor is as low as 0.11 μg/mL in the detection of the phosphate-buffered saline sample, and the spike-and-repetition rate is 105.56% in the detection of the real serum sample, which partly shows the practicability of the biosensor. This indicates that the LR-SPR biosensor provides better response compared with existing similar sensors and can be regarded as a valuable method for biochemical analysis and disease detection.

## 1. Introduction

Surface plasmonic resonance (SPR) biosensors based on optical fiber have been widely studied in immunoassay, analytical chemistry and disease examination due to their outstanding properties of compact size, anti-electromagnetic interference, high sensitivity and label-free detection. However, traditional fiber based SPR sensors have limited ability in further reducing the limit of detection (LOD) because the relatively shallow penetration of the evanescent field prevents the effective detection of the refractive index variation of biomolecules. Furthermore, the large full width at half-maximum (FWHM) and the low figure of merit (FOM, the ratio between the sensitivity of the sensor and the FWHM) in the fiber SPR-sensing spectrum have seriously restricted the resolution and accuracy of the detection. Therefore, methods for improving the performance of SPR sensors have been widely investigated. There are mainly two categories. In the first category, zero-dimensional materials such as noble metal nanoparticles [1], one-dimensional materials such as halloysite nanotubes [2] and two-dimensional materials such as black phosphorus [3] are applied to modify the sensor surface. These materials are used to concentrate target biomolecules, to promote the transmission of electrons between the metallic layer and nanomaterials, and to strengthen the confined electric field. All these can enhance the sensitivity of the sensor. The second branch is to shape the induced electric response by changing the dielectric film structure of the sensor to improve the sensing performance. Corresponding SPR effects include localized-SPR (L-SPR) [4,5], long-range SPR (LR-SPR) [6], coupled plasmonic waveguide resonance [7] and waveguide-coupled SPR [8]. Among these, LR-SPR has attracted more and more attention due to its high sensitivity and narrow FWHM [6].

As for the fiber-based SPR sensor, similar values between the refractive index in the fiber and that in the analyte are key factors affecting the sensitivity. Fiber is generally made of silica with a refractive index from 1.45 to 1.50. The sensitivity of the sensor is higher in the detection of high-refractive index substances such as crude oil asphaltene [9], while the sensitivity is lower in the detection of low-refractive index substances such as phosphate-buffered saline (PBS)-prepared biological solutions with concentrations near zero. LR-SPR is a type of collective oscillation of free electrons in the noble metal stimulated by adding a lossless matching layer (LML, of which the dielectric constant is a pure real value and the refractive index is lower than the substratum) between the substratum and the metallic layer. As a buffer layer, the LML increases the similarity level between the refractive index in the fiber and the refractive index in the low-concentration biochemical solution, and reduces the loss of surface plasmonic waves (SPWs) [6]. Thus, the sensitivity of the sensor is enhanced and the FWHM becomes narrower. In this case, it will be easier to find the peak and to improve the detection accuracy.

It is promising to develop new type of fiber SPR sensor based on the combination of the electric field coupling produced by zero-dimensional nanomaterials and the LRSPR effect. In order to realize the trace detection of biomolecules, the double-antibody sandwich immunoassay (antibody 1-antigen-antibody 2) can be introduced into the development of the SPR sensor. In 2020, Yu et al. [10] utilized an SPR-based double-antibody sandwich approach to reduce the LOD of miRNA-125b down to 123.044 pM. In 2021, Belen et al. [11] realized the picomolar level detection of staphylococcal enterotoxin G using a similar approach. The double-antibody sandwich increases the coverage density on the surface of the SPR sensor to generate a stronger response signal. Meanwhile, sensors based on new electromagnetic resonance effects (e.g., lossy mode resonance (LMR) [12], Fano resonance [13], surface lattice resonance [14]) and novel substrates such as long period grating in double cladding fiber [15] are also developed for the biochemical sensing.

According to the above discussions, a LR-SPR biosensor is designed based on the synergistic improvement of double-antibody sandwich immunoassay and plasmonic coupling. On the one hand, the double-antibody sandwich assay can increase the molecular surface density in the sensor [16]. On the other hand, the coupling between plasmons can lead to an obvious enhancement in the localized electromagnetic field and a significant perturbation in the evanescent field. This will increase the penetration depth of the evanescent field in the surrounding medium and make the sensor be more sensitive to the change of surrounding refractive index [1,6]. The synergy of the two aspects can significantly amplify the SPR signal, to improve the sensitivity of the sensor and to reduce the LOD. Moreover, the 350 nm absorption line provides the self-compensation function for the physical disturbance in the detection. In this paper, human-immunoglobulin G (H-IgG), which is the main substance of anti-infective immunity in human body, has been employed as the analyte. The large core side polishing fiber (SPF) has been used as the substratum to stimulate the LR-SPR and the L-SPR effects. Our developed sensor is small in size, low in cost, easy to form the distributed sensing and can realize remote real-time online detection for H-IgG with low LOD and high accuracy and reliability.

## 2. Sensing Principle and Simulation Analysis

**Figure 1:**
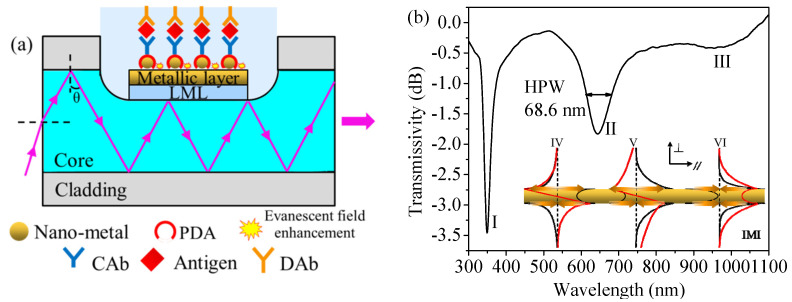
(**a**) Diagram of the LR-SPR biosensor based on synergistic improvement of sandwich immunoassay and plasmonic coupling. (**b**) Transmission spectrum of the LR-SPR sensor (I: characteristic absorption line, II: long-range resonance curve, III: short-range resonance curve). Inset: the field/current distributions of three modes with the lowest loss in the insulator-metal-insulator (IMI) model (IV–VI: three modes, red line: electric field distribution, black line: magnetic field distribution, orange arrow: current conduction). Reproduced with permission from reference [21] by John Wiley and Sons.

As shown in Figure 1a, the essential film structure of the LR-SPR sensor includes SPF, LML, metallic layer and target analyte. Since the metallic layer is sandwiched by two dielectric layers with similar refractive indices, SPWs are generated on both sides of the metallic layer. According to the coupling mode theory [17], the energy of transmission light is coupled into two rows of SPWs. The two SPWs will interfere with each other. The constructive interference forms long-range SPWs (LR-SPWs) and the destructive interference forms short-range SPWs (SR-SPWs). These effects excite LR-SPR and short-range SPR [18,19,20], respectively. Therefore, the transmission spectrum of the LR-SPR sensor contains a long-range resonance curve and a short-range resonance curve, as shown in Figure 1b. Since the field distribution of the LR-SPWs [21] shown in Figure 1b is mainly concentrated in the dielectric layers, which indicates a weaker restriction on the vertical axis and a lower loss on the parallel axis, the penetration depth of LR-SPWs in the analyte is larger (in the order of micrometers) and the propagation distance of LR-SPWs on the interface between the metallic layer and the medium layer is longer. Therefore, the long-range resonance curve has narrower FWHM and higher FOM, and also has more significant advantages in detecting biological macromolecules compared with traditional SPR sensors [18,19]. The energy of SR-SPWs is mainly focused on the metallic layer surface, and thus it can be used for the monoatomic layer detection [22]. The short-range resonance curve may disappear due to the optimized design of the film thickness.

**Figure 2:**
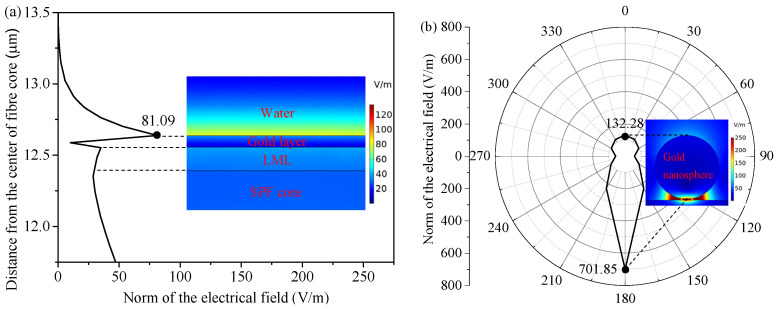
(**a**) Axial electric field distribution of the LR-SPR sensor and (**b**) electric field distribution of the gold nanosphere wall at different angles in the gold nanosphere-modified LR-SPR sensor. Inset: mode field distribution of the LR-SPR sensor and the gold nanosphere-modified LR-SPR sensor.

Modifying the nanometal to the gold layer surface in the sensor can further enhance the sensitivity, as shown in Figure 1a. The finite element analysis shown in Figure 2 suggests that the electric field intensities at the top and the bottom of the gold nanospheres in the gold nanosphere-modified LR-SPR sensor are, respectively, 1.63 times (81.09–132.28 V/m) and 8.66 times (81.09–701.85 V/m) higher than those of the bare LR-SPR sensor surface, respectively. This is because the near-field electron coupling between the localized surface plasmon, originating from the nanometal, and the long-range surface plasmon, arising from the metallic layer, leads to a stronger evanescent field perturbation [6]. The sensitivity is related to the overlap integral of the electrical intensity in the analyte region [23], thus the gold nanosphere-modified LR-SPR sensor possesses higher sensitivity. The specific antigen can be detected by modifying the capture antibody (CAb) on the nonmetal surface. The parameter that the sensor can detect is the average refractive index of all substances on its surface. In order to enhance the detection sensitivity, detection antibody (DAb) can be added after the antigen, to build the double-antibody sandwich immunoassay (i.e., CAb-antigen-DAb structure, shown in Figure 1a) [16,24]. This can increase the change of the refractive index in the medium layer on the sensor surface.

**Figure 3:**
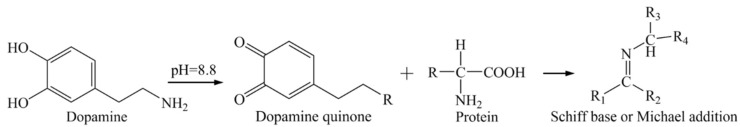
Mechanism of dopamine immobilized antibody.

The LML used in this paper can absorb the light field near a wavelength of 350 nm. This produces a characteristic absorption line in the transmission spectrum of the sensor, as shown in Figure 1b. When the sensor is disturbed, the whole transmission spectrum could shift, but the wavelength difference between the resonance curve and the absorption line remains unchanged. Detection results can be obtained based on the stable wavelength difference rather than the single resonance curve. Therefore, the sensor possesses the in situ self-compensation function which allows corrections for errors of spectral drifts induced by the mechanical vibration and other disturbances [25]. Polydopamine (PDA), as a polymer material, can realize the controllable self-polymerization and possesses excellent biocompatibility and hydrophilicity. The strong adhesion (similar to the mussel mucus) and the strong hydrolysis resistance of the PDA make it more suitable as a biosensing matrix than the 11-mercaptoundecanoic acid. Therefore, PDA has been used as an antibody coupling agent, and the specific principle is shown in Figure 3. The ortho-diphenol functional group of dopamine will be converted into quinone group under the weakly basic conditions. Quinones can be covalently coupled with amino- or thiol-terminated biomolecules by Schiff base and Michael addition reaction [16].

## 3. Materials and Methods

### 3.1. Materials and Reagents

The multimode fiber with core/cladding diameters of 105/125 µm and a numerical aperture of 0.22 is purchased from Beijing Scitlion Technology Corp., Ltd. (Beijing, China), to fabricate the SPF. Terbium acetate and hexafluoroacetylacetone are purchased from J&K Scientific Corp., Ltd. (Beijing, China) to prepare the LML solution. 1,4-dithiothreitol and gold nanosphere aqueous solution (diameter: 50 nm) are purchased from Sigma-Aldrich LLC. (St. Louis, MO, USA). Dopamine hydrochloride, Tris buffer (pH = 8.7, 10 mM), PBS (pH = 7.2, 10 mM), H-IgG (contains four isoforms: IgG 1–4), rabbit anti H-IgG, mouse anti H-IgG, bovine serum albumin (BSA), horse IgG, goat IgG and dog IgG are purchased from Wuhan Huamei Biotech Corp., Ltd. Wuhan, China.

### 3.2. Manufacturing of Sensing Probe

**Figure 4:**
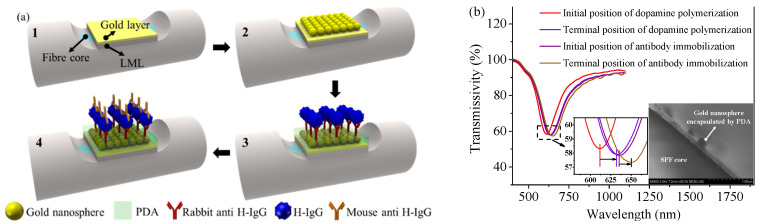
(**a**) Sensing probe manufacturing process. (**b**) Resonance spectrum changes of the sensor after PDA self-polymerization and antibody immobilization. Inset: SEM image of the cross-section of the LR-SPR sensor modified with gold nanospheres and PDA.

The manufacturing process of the sensing probe is described as follows. 1.18 g of Tb (III) acetate is firstly dissolved in 20 mL of deionized water at 0 °C. Then, 1.50 mL of hexafluoroacetylacetone is drop wise added to the above solution. The mixture will produce white-green precipitations after stirring for 180 min. Finally, precipitations are filtered and recrystallized from distilled water to produce acicular crystals. The LML solution is obtained by dissolving the crystals in the absolute ethanol. A 100 nm LML with a refractive index of approximately 1.365–1.400 [26,27] is coated on the surface of the polishing area of the SPF (polishing depth: 50 μm, polishing length: 20 mm) by a dip-coater (SYDC-200, Shanghai SanYan Technology Corp., Ltd. China, Shanghai, China). A 40 nm gold layer is then coated using a magnetron sputtering machine (MSP-3200, Beijing Chuangshiweina Technology Corp., Ltd. China, Beijing, China) to complete Step 1 in Figure 4a.

The sensor in Step 1 is covalently coupled with a layer of gold nanospheres through additional gold-sulfur bonds to complete Step 2. The sensor in Step 2 is immersed in dopamine solution (2 mg/mL, pH = 8.8) and constantly shaken to form a self-polymerized dopamine layer. The resonance spectrum of the sensor in Figure 4b is monitored during the self-polymerization process, and the resonance curve shows significant redshift and broadening, indicating that the sensitivity is enhanced but the detection accuracy is decreased. Experimental results show that the sensor performs better when the self-polymerization time is 30 min. The scanning electron microscope (SEM) image of the cross-section of the sensor is shown in Figure 4b. The sensor was dried and was then immersed in 200 μg/mL rabbit anti H-IgG solution. The whole component was incubated overnight at 4 °C to fully immobilize the antibody. In order to investigate whether the antibody is immobilized to the sensor surface, the resonance spectrum of the sensor is monitored as shown in Figure 4b when the sensor is immersed in the antibody solution. It is found that the resonance curve appears obvious redshift which indicates that the antibody is effectively immobilized. Additionally, BSA has been used as blocking agent in surface passivation.

### 3.3. Experimental Setup

**Figure 5:**
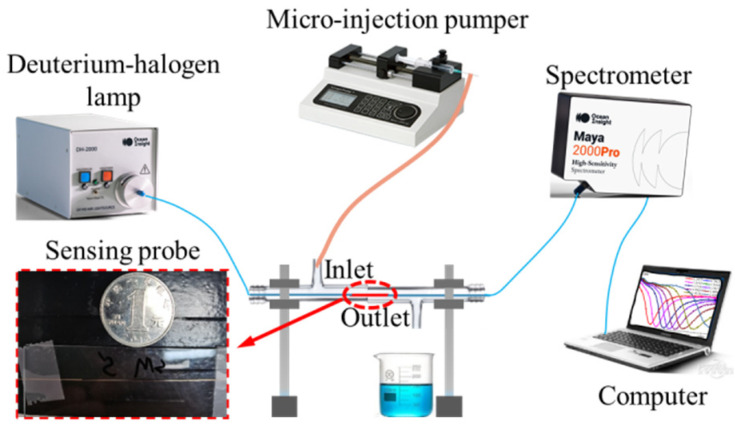
Schematic of the experimental setup.

The sensor is used as a sensing probe to connect to the experimental setup shown in Figure 5. The light emitted by the wide-spectrum lamp (DH-2000-BAL, Ocean Insight, Inc., Orlando, FL, USA) passes through the unpolished fiber into the sensing probe area located in the customized glass tube. The transmitted light is received by the spectrometer (Maya2000 Pro, Ocean Insight, Inc., Orlando, FL, USA), and the transmission spectrum is displayed on the computer. The H-IgG solution with specific concentration is injected into the glass tube via a micro-injection pumper (LSP01-3A, Longer Precision Pump Corp., Ltd. Baoding, China) through the inlet to complete Step 3, and the excess solution flows out to the waste cylinder through the outlet. The reaction lasted for 40 min after the injection of H-IgG solution. Then, the mouse anti H-IgG solution with a concentration of 40 μg/mL is injected to complete Step 4 with a reaction lasting 15 min. To detect other concentrations of H-IgG, the sensor can be rinsed several times with PBS and the above steps will be repeated. Note that the above immunoreaction has been carried out in a room with a constant temperature of 25 °C.

## 4. Results and Discussions

### 4.1. Refractive Index Sensing Experiment

**Figure 6:**
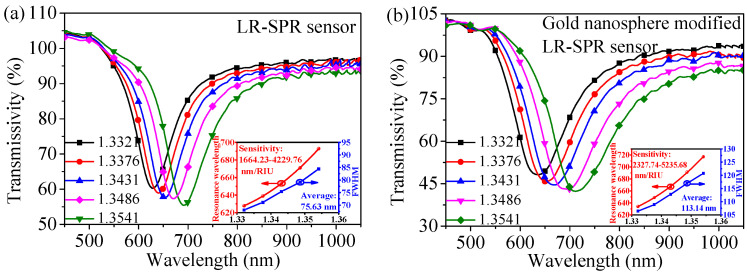
The resonance spectra of the (**a**) LR-SPR sensor and (**b**) gold nanosphere-modified LR-SPR sensor. Inset: red curve represents the binomial fitting of refractive index and resonance wavelength, and the tangent slope at each point represents sensitivity, and blue curve represents the FWHM variation with different refractive index.

To investigate the influence of plasmonic coupling on the sensor performance, the refractive index sensing performance of the bare LR-SPR sensor and the gold nanosphere-modified LR-SPR sensor have been explored. Experimental results are shown in Figure 6 and Table 1. The modification of gold nanospheres increases the surface molecular weight in the sensor and strengthens the intensity of localized electromagnetic field. Therefore, the resonance curve position of the gold nanosphere-modified LR-SPR sensor exhibits redshift, and the average sensitivity shows an enhancement of 838.28 nm/RIU (2946.44–3784.72 nm/RIU) compared with that of the bare LR-SPR sensor. Due to the light scattering characteristics of gold nanospheres, the average FWHM of the gold nanosphere-modified LR-SPR sensor is increased by 37.51 nm (75.63–113.14 nm) compared with that of the bare LR-SPR sensor. For both types of sensors, the resonance curves will be gradually broadened with the increase in the refractive index. This is because the number of modes, which excite the SPR, increases. In other words, the phase-matching conditions for exciting SPR become less stringent.

### 4.2. Human IgG Detection

#### 4.2.1. PBS Matrix Sample Detection

**Figure 7:**
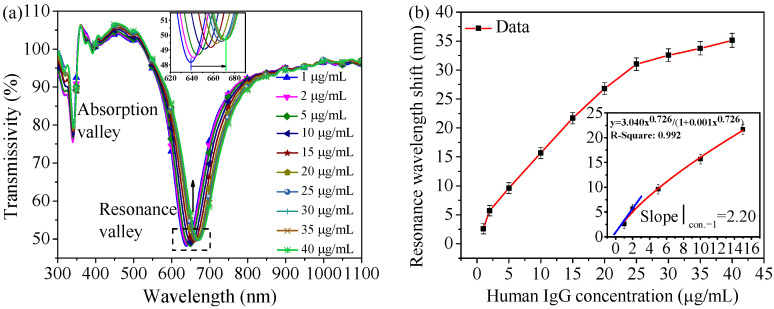
(**a**) Transmission spectrum obtained by the sensor detecting H-IgG solutions with different concentrations. (The position of each resonance curve is the stable position when detecting the corresponding H-IgG solution.) (**b**) Resonance curve shift corresponding to the detection of H-IgG solutions in various concentrations. Error bar = ±S.D., and the same assay in the same experimental conditions is repeated three times. Inset: calculation of sensor’s sensitivity.

The developed sensor has been used to detect PBS-prepared H-IgG solutions with different concentrations, and detection results are shown in Figure 7. In the process of combining the two antibodies and antigens to form the macromolecular complex, the average refractive index of the sensor surface increases continuously, and the resonance curve exhibits redshift, as shown in Figure 7a. In addition, the self-compensation function of the sensor improves the detection reliability. Applying the Langmuir fitting to the low concentration part of the curve in Figure 7b, the tangent slope at the first point (1 μg/mL) is considered as the sensor’s sensitivity $S_{n\lambda }$ and its value is 2.20 nm/(μg/mL). The wavelength resolution of the experimental system is 0.24 nm according to the 2ρ principle [28]. The LOD of the sensor is 0.109 μg/mL according to Equation (1) [29,30].

(1) $$LOD=2\rho /S_{n\lambda }$$

where ρ is the standard deviation (S.D.) obtained from the repeated measurement of the resonance wavelength in the sensor corresponding to a specific refractive index every 30 s, according to Equation (2).

(2) $$\rho =\sqrt{\frac{\sum_{i=1}^{n}{\left(\lambda_{i}-\bar{\lambda }\right)}^{2}}{n-1}}$$

where $\lambda_{i}$ and $\bar{\lambda }$ are the resonance wavelength value measured each time and the average value of resonance wavelength, respectively. i=1,2,3,…,100, n=100.

The LOD of 0.109 μg/mL is obviously lower than the expected range of H-IgG concentration in the blood plasma or serum (4.07 × 10^3^–2.17 × 10^4^ µg/mL [31]). This can effectively reduce the influence of measurement errors on detection results. In addition, the principle of the developed sensor works based on the immune reaction between the antigen and the antibody. Other types of antigens can also be detected when corresponding antibodies are employed. Therefore, biomarkers with lower concentrations in the blood plasma or serum such as prostate specific antigen [32] can be detected by utilizing the SPR sensor with a lower LOD.

In fact, the LOD of the sensor is related to the wavelength resolution of the spectrometer, the detectable concentration range, the data processing and many other factors. IgG detection results using the electromagnetic resonance sensor have also been listed for similar methods and analytes, as shown in Table 2. It indicates that results in this paper have achieved good agreements with the reported research works.

**Figure 8:**
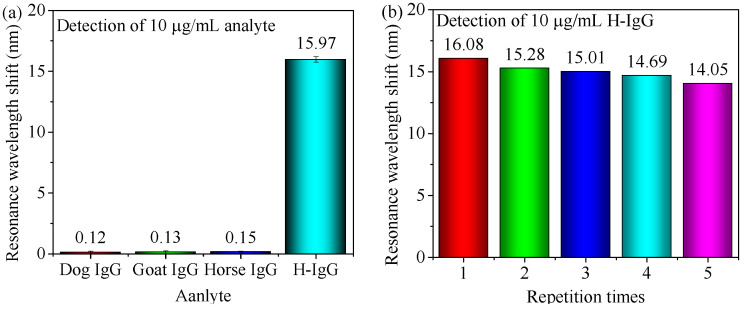
The (**a**) specificity and (**b**) repeatability of the sensor. Error bar = ±S.D., and the same assay in the same experimental conditions is repeated three times.

Different protein solutions with the same concentration of 10 μg/mL are also detected using this developed sensor. H-IgG solutions with the same concentration of 10 μg/mL have been detected multiple times. As Figure 8a shows, the resonance curve exhibits obvious redshift in the detection of H-IgG because the specific binding only occurs between the antibody and H-IgG. As shown in Figure 8b, the resonance wavelength redshift does not change much and gets slightly smaller after five cycles because the amount of antibody decreases during the dissociation and the rinse of the sensor. Above-experimental results indicate that the sensor has good specificity and repeatability. Meanwhile, the experimental setup exhibits good reproducibility.

#### 4.2.2. Serum Matrix Sample Detection

**Figure 9:**
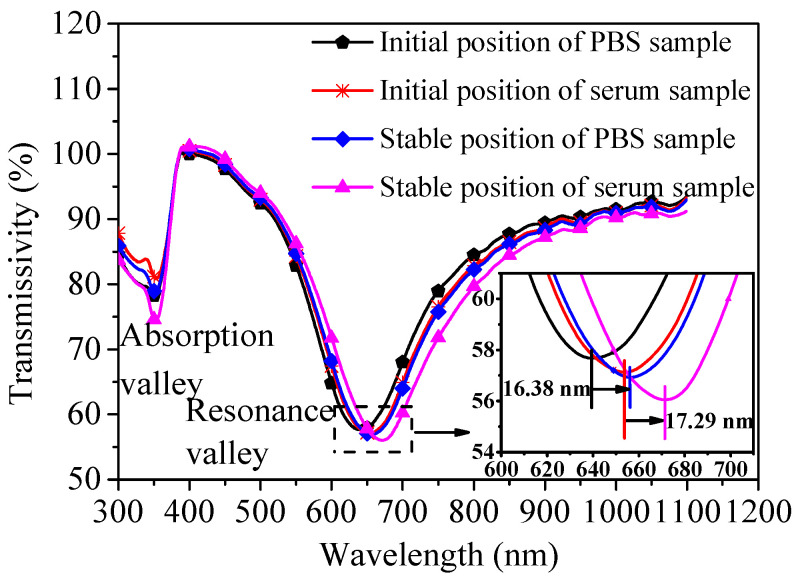
Resonance spectrum of the sensor obtained in detecting PBS sample and serum sample.

Controlled experiment has also implemented to investigate the practicability of the developed sensor. 10 μg/mL H-IgG solutions are separately prepared by PBS and donkey serum. The sensor is used to detect the PBS matrix sample at first, and the resonance curve shift $\Delta \lambda_{PBS}$ is 16.38 nm. The sensor is then rinsed and is used to detect the serum matrix sample, and the observed resonance curve shift $\Delta \lambda_{serum}$ is 17.29 nm. It is worth noting that the initial positions of the resonance curve are different for the two test samples, because the refractive indices of the two samples are different. Corresponding experimental results are shown in Figure 9. Repetition rate is defined according to Equation (3) [24].

(3) $$\mathrm{Repetition}\;\mathrm{rate}\;=\;\Delta \lambda_{serum}/\Delta \lambda_{PBS}$$

The calculated repetition rate is 105.56%, which is close to 100%. This indicates that the nonspecific interaction induced by donkey serum matrix is acceptable, and it is feasible for the developed sensor to detect the real serum sample. For comparison, Table 3 lists the repetition rate obtained from our research and reported works using similar methods, which suggests the repetition rate achieved in this article is at a good level.

## 5. Conclusions

A novel approach for introducing the double-antibody sandwich immunoassay and plasmonic coupling into the LR-SPR sensor has been reported and applied to the detection of H-IgG. The developed biosensor is used to detected the PBS matrix H-IgG sample with different concentrations, and the demonstrated sensitivity and LOD reach 2.20 nm/(μg/mL) and 0.11 μg/mL, respectively. The remarkable amplification of sensing response is attributed to: (1) the stronger local electromagnetic field obtained from the combination of LR-SPR and L-SPR, and (2) the more obvious refractive index change of the dielectric layer on the sensor surface obtained by the double-antibody sandwich immunoassay. The biosensor has also been employed to detect the PBS matrix sample and the serum matrix sample (with the same concentration), and the ratio of the resonance curve shift obtained in the detection is 105.56%. This indicates that the nonspecific binding induced by the serum matrix puts negligible effect on the sensing performance and the developed sensor possesses certain practicability. Furthermore, the self-compensation against the disturbance allows the developed biosensor to provide higher reliable detection results. In future research, antigen can be labeled on the surface of the gold nanosphere to bind with the CAb attached to the gold layer. Therefore, the antigen-antibody binding will occur in the gap between the gold nanosphere and the gold layer, and the enhancement of the localized electromagnetic field will be fully utilized. In order to further reduce the LOD of the sensor, two-dimensional nanomaterials with high refractive indices can also be introduced.

## Figures and Tables

**Table 1:** The sensitivity of the two types of LRSPR sensor.

Refractive Index	Sensitivity of LR-SPR Sensor (nm/RIU)	Sensitivity of Gold Nanosphere-Modified LR-SPR Sensor (nm/RIU)	Enhancement (nm/RIU)
1.3321	1664.23	2327.74	663.51
1.3376	2303.42	3056.39	752.97
1.3431	2946.92	3786.73	839.81
1.3486	3587.85	4517.07	929.22
1.3541	4229.76	5235.68	1005.92
Average	2946.44	3784.72	838.23

**Table 2:** Comparison of the research results in this paper with those reported in recent years.

Principle	Method	Analyte	Concentration Range (μg/mL)	LOD (μg/mL)	Refs.
SPR	Gold layer/MoSe_2_	Goat-anti-Rabbit IgG	10–100	0.33	[29]
	Gold layer	Goat-anti-Mouse IgG	0.50–10	0.10	[33]
	Gold layer/PDDA-PSS	Human IgG	25–1000	1.75	[34]
L-SPR	Gold nanoparticles arrays	Human IgG	1–100	0.16	[4]
	MoS_2_/Gold nanoparticles	Human IgG	6.26–626.32	0.62	[5]
LMR	SnO_2−x_	Goat-anti-Mouse IgG	1–40	0.60	[35]
	ITO	Goat-anti IgG	0.02–1.53	-	[36]
LR-SPR	MgF_2_/Gold layer	Human IgG	0.3125–10	0.0032	[28]
	LML/Gold layer/Gold nanoshells	Human IgG	1–40	0.20	Previous work [37]
	Sandwich immunoassay and plasmonic coupling			0.109	This work

**Table 3:** Comparison between the repetition rate in this paper and those reported works.

Method	Analyte	Spiked (μg/mL)	Repetition Rate (%)	Deviation from 100% (%)	Refs.
Gold layer/hollow gold nanospheres (HGNPs)/PDA wrapped magnetic carbon nanotube	Human cardiac troponin	0.01	112.50	+12.50	[16]
		0.08	106.90	+6.90	
		0.30	97.01	−2.99	
Gold layer/HGNPs/PDA-Ag@Fe_3_O_4_-rGO	Rabbit IgG	0.075	126.09	+26.09	[24]
		0.60	115.38	+15.38	
		5.00	89.23	−10.77	
Gold layer/Fe_3_O_4_-HGNPs	Human IgG	0.03	116.67	+16.67	[38]
		0.125	110.34	+10.34	
		0.50	106.84	+6.84	
PDA/AgNPs/PDA/gold layer	Horse IgG	5	107.14	+7.14	[39]
		10	111.11	+11.11	
		20	108.33	+8.33	
LML/gold layer/gold nanoshells	Human IgG	15	107.62	+7.62	Previous work [37]
Sandwich immunoassay and plasmonic coupling	Human IgG	10	105.56	+5.56	This work

## Data Availability

The data presented in this study are available on request from the corresponding author. The data are not publicly available due to personal data protection.
